# A251 EOSINOPHILIC ESOPHAGITIS IN AN END-STAGE ACHALASIA PATIENT

**DOI:** 10.1093/jcag/gwae059.251

**Published:** 2025-02-10

**Authors:** S Pan, K Woodman

**Affiliations:** Undergraduate Medicine, McMaster University, Hamilton, ON, Canada; Undergraduate Medicine, McMaster University, Hamilton, ON, Canada

## Abstract

**Background:**

Achalasia is a rare motility disorder involving abnormal esophageal motility and impaired lower esophageal sphincter relaxation. A growing number of reports have identified esophageal eosinophilia levels consistent with eosinophilic esophagitis (EoE) in achalasia patients, suggesting a possible pathophysiological association between both conditions that may yield clinical significance for diagnosis and treatment.

**Aims:**

The aim of the present report is to highlight this association, through the case of a Type 1 Achalasia patient whose mid-esophageal biopsies were significant for concurrent EoE.

**Methods:**

The case and its associated figures were compiled from the patient’s medical records following informed consent.

**Results:**

A 38-year-old gentleman was referred to our motility service for manometric assessment. The patient suffered from a 4-5 year history of persistent, progressively worsening dysphagia, recurrent regurgitation and weight loss. Endoscopic assessment prior to assessment by our service was significant for EoE. Manometry patterns were consistent with Type 1 achalasia, though it was unclear whether this had developed prior to or after the patient’s EoE. The patient’s symptoms continued to progress in the months after his achalasia diagnosis, despite proton pump inhibitor therapy, steroid therapy and an elimination diet. He was ultimately referred to the General Surgery service in Hamilton, Ontario for completion of a Heller Myotomy. The post-operative course was uncomplicated, and he was able to return to an oral diet shortly afterwards, with some lingering dyspepsia but no further dysphagia or regurgitative symptoms.

**Conclusions:**

The present case supports pre-existing reports in the literature that achasia and EoE can happen concurrently, and that a pathophysiological correlation may be involved, notably regarding the inflammatory processes in EoE and how they might contribute to esophageal neuronal damage. Continued research is needed to better understand this relationship.

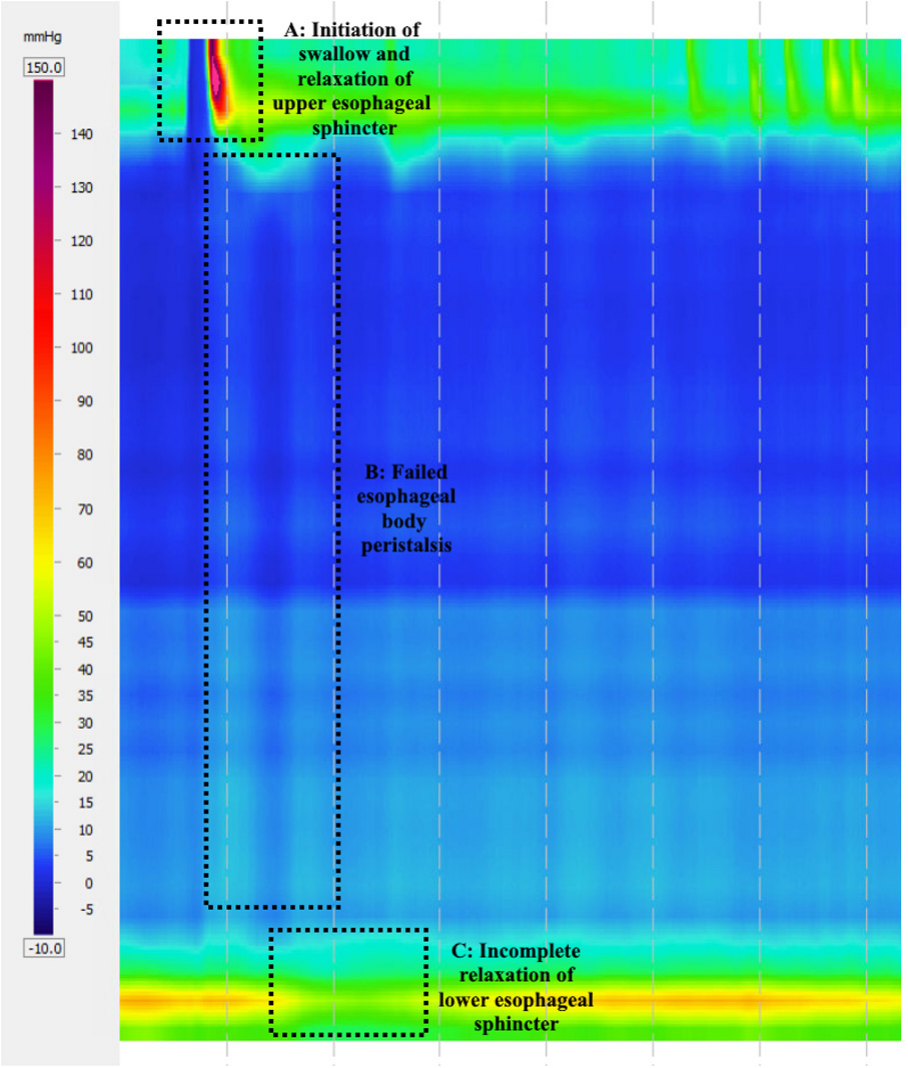

Esophageal pressure topography plots taken from high-resolution manometry performed on April 18, 2023, representing the average pressure patterns of successful swallows during the endoscopic procedure. Highlighted is the relaxation of the upper esophageal sphincter during initiation of the swallow (A), along with the absence of peristaltic pressures along the length of the esophagus (B) in the setting of incomplete deglutitive relaxation of the lower esophageal sphincter (C), consistent with type 1 achalasia.

**Funding Agencies:**

None

